# Pathogenicity and virulence of Hepatitis B virus

**DOI:** 10.1080/21505594.2022.2028483

**Published:** 2022-01-31

**Authors:** Yu-Chen Chuang, Kuen-Nan Tsai, Jing-Hsiung James Ou

**Affiliations:** Department of Molecular Microbiology and Immunology, University of Southern California Keck School of Medicine, Los Angeles, CA 90089, USA

**Keywords:** Hepatitis B virus, HBV genomic organization, HBV lifecycle, HBx signaling, viral pathogenesis, hepatocarcinogenesis, interferon immune responses, HBV persistence, antibody-dependent cell-mediated cytotoxicity

## Abstract

Hepatitis B virus (HBV) is a hepatotropic virus and an important human pathogen. There are an estimated 296 million people in the world that are chronically infected by this virus, and many of them will develop severe liver diseases including hepatitis, cirrhosis and hepatocellular carcinoma (HCC). HBV is a small DNA virus that replicates via the reverse transcription pathway. In this review, we summarize the molecular pathways that govern the replication of HBV and its interactions with host cells. We also discuss viral and non-viral factors that are associated with HBV-induced carcinogenesis and pathogenesis, as well as the role of host immune responses in HBV persistence and liver pathogenesis.

## Introduction

Hepatitis B virus (HBV) is a hepatotropic virus that can cause severe liver diseases including acute and chronic hepatitis, cirrhosis and hepatocellular carcinoma (HCC). It was first detected in the serum of Australian aborigines during the 1960s [1]. The newly discovered antigen at that time, initially named “Australia antigen” and presently called HBV surface antigen (HBsAg), was frequently observed in leukemia patients. It has since become clear that hepatitis B is a widespread disease, and more than 2 billion people in the world have been exposed to HBV. The World Health Organization (WHO) estimated that 296 million people were chronically infected by HBV in 2019 (https://www.who.int/news-room/fact-sheets/detail/hepatitis-b). In endemic areas such as in Sub-Saharan Africa and Asia-Pacific countries, the mother-to-child transmission at birth, also known as vertical transmission, is the most common way of transmission. People who acquired HBV early in life would often become chronic carriers of the virus without therapeutic intervention. HBV can also be transmitted via sex or contaminated needles during injection drug use. The transmission of HBV between adults, also known as the horizontal transmission, usually leads to self-limited acute infection.

HBV is a hepatotropic virus that belongs to the family of *hepadnaviridae*. It has a narrow host range and infects only humans and a few other primate species. HBV is spherical in shape with a diameter of 42 nm. The mature and infectious HBV particle is also called Dane particle[2]. HBV has a lipid envelope, which can be removed by non-ionic detergents to expose the ~27-nm viral core [3,4]. The core particle, also known as the capsid particle, contains an endogenous DNA polymerase activity and a circular and partially double-stranded DNA with a length of approximately 3.2 kilobases (kb) [5–7]. The viral DNA polymerase also has the reverse transcriptase (RT) activity. In addition, a kinase activity is also detected inside the core particle[8]. There are three related viral envelope glycoproteins named large (L), middle (M), and small (S) HBsAg, which are also known as preS1, preS2 and major S proteins, respectively. These three envelope proteins are inserted in the envelope of the virion at a mass ratio of approximately 3:2:5 or a molar ratio of 1:1:4[9]. The core particle displays the core antigenic determinant known as the core antigen (HBcAg). It consists of 90 or 120 core protein dimers[9], which form the shell of the core particle. A mature HBV virion has a buoyant density of 1.24 to 1.26 g/cm^3^.

During infection, complete and incomplete HBV viral particles are released into the serum of patients. Complete HBV particles are the infectious Dane particles and their titers in the blood can be as high as 10^9^ genome copies per ml. In contrast, incomplete viral particles are subviral particles (SVPs) consisting of mostly 22-nm spherical and filamentous particles, which are noninfectious and can reach up to 10^14^ particles per ml^11^. SVPs consist of only the surface proteins and host-derived lipids and lack the core particle. The 22-nm spherical particles contain primarily M and S surface proteins with a mass ratio of 1:4 and a small amount of the L protein, while the 22-nm filamentous particles contain L, M, and S surface proteins with a mass ratio of roughly 1:1:4. This class of SVPs has a buoyant density of 1.18 g/cm^3^. The biological function of this enormous amount of SVPs in the patient serum is unclear. The possibility that they may serve as decoys to sequester host neutralizing antibodies had been proposed [10,11].

Another class of circulating viral particles is the empty virions, which contain the viral envelope and viral capsid but not the genome. Empty virions are found at a level of up to 10^11^ particles per ml of blood in infected patients and have a density of 1.20 g/cm^3^, lower than that of the complete virion[12]. Other noninfectious viral particles are also detected in the blood, including the particles containing viral RNA termed as RNA virions and the hard-to-detect nonenveloped capsids[13].

## HBV genomic organization

**Figure 1.**
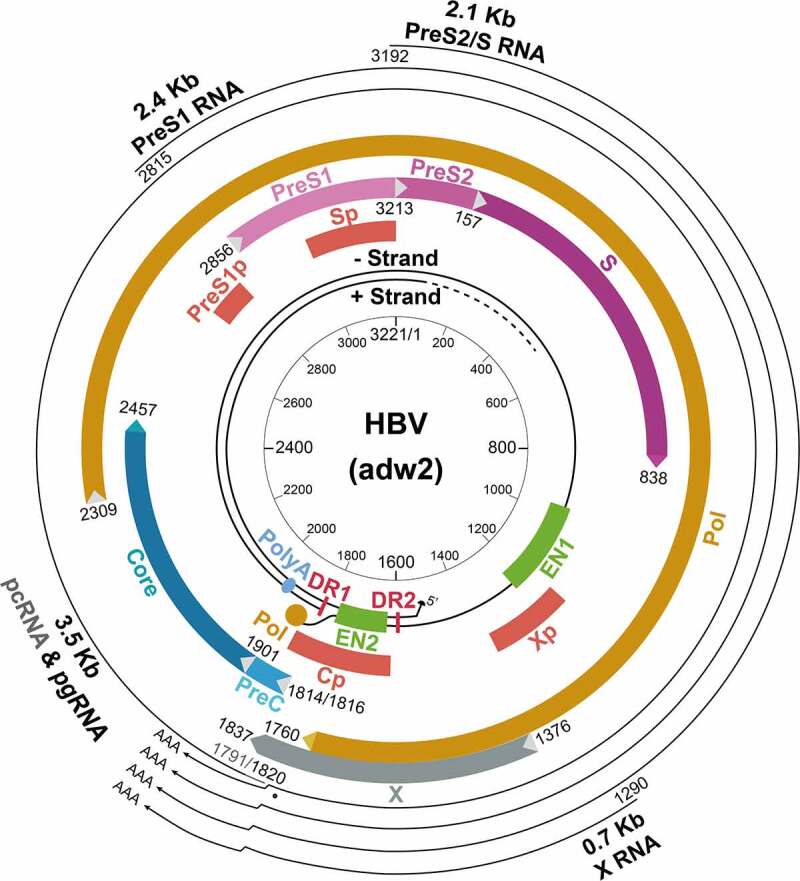
**The structure of the HBV genome**. The locations of the regulatory elements including EN1 and EN2 enhancers and the four promoters are shown. The HBV RNA transcripts, their approximate transcription initiation sites, and the unique poly(A) site are also indicated. The locations of the four ORFs are shown. The unique EcoRI cutting site is defined as nucleotide (nt.) 1. The nt. number is based on the HBV adw2 isolate (genotype A). Cp, core promoter; PreS1p, preS1 promoter; Sp, S promoter; Xp, X promoter.

HBV has a small DNA genome, which is a partially double-stranded and relaxed circular DNA (rcDNA) molecule (Figure 1). This asymmetrical genomic structure has a minus strand covering the whole genome and an incomplete plus strand with variable 3’ ends [7,14]. The minus strand has a terminal protein, which is the viral DNA polymerase, covalently linked to its 5’ end through a tyrosyl-DNA phosphodiester bond. In contrast, the plus strand has a short 5’-end capped RNA fragment[15]. The circular configuration of the genome is maintained by these two DNA strands through base pairings[16]. The minus-strand DNA synthesis is primed by its terminal protein, whereas the plus-strand DNA synthesis is primed by the short RNA fragment located at its 5’-end. These two primers (i.e., the terminal protein and the short RNA fragment) remain linked to their respective DNA strands after viral DNA synthesis. Details of the viral DNA replication will be discussed later in this article.

The viral genome contains four overlapping open reading frames (ORFs). It also contains four promoters, two enhancer elements (EN1 and EN2), and a single polyadenylation site for viral RNA transcription, and several *cis*-acting signals for DNA replication (Figure 1). The four ORFs in the minus strand, named P, S, C and X, encode the DNA polymerase, HBsAg proteins, core and precore proteins, and the X protein (HBx), respectively. The analysis of HBV RNA transcripts in HBV-infected hepatocytes revealed four primary transcripts with the lengths of 3.5-kb, 2.4-kb, 2.1-kb and 0.7-kb, corresponding to the mRNAs of precore and core proteins, large surface protein, middle and small surface proteins, and HBx. These transcripts are transcribed from the four different promoters, but they share the same polyadenylation site in the C ORF and thus have the same 3’ end. The 3.5-kb RNA contains two separate transcripts with nearly identical length. These two RNA transcripts are the shorter pregenomic RNA (pgRNA) and the longer precore RNA (pcRNA), which differ in length by approximately 30 nucleotides. pgRNA is the mRNA of the core protein and the polymerase and also the template for the replication of the HBV genomic DNA [17]. In contrast, pcRNA is the mRNA of the precore protein, which is the precursor of the hepatitis B e antigen (HBeAg) found in the sera of HBV patients[18].

The C gene ORF has two in-frame start codons. The translation initiating from the upstream start codon produces the 25-kDa precore protein (p25) and the translation from the downstream start codon generates the 21-kDa core protein (p21). The P gene ORF is the largest ORF, accounting for nearly 80% of the viral genome and overlapping with all other ORFs. It encodes the 90-kDa viral DNA polymerase, which contains four domains. These domains are, from the N-terminus to the C-terminus, the terminal protein (TP) domain, which is the primer for the minus-strand DNA synthesis, a “spacer” domain, a catalytic reverse transcriptase (RT) domain, and a RNase H domain. The S gene ORF encodes the three HBsAg proteins. It overlaps with the spacer and RT domains of the P gene ORF. The S gene ORF contains three in-frame start codons that divide the S gene ORF into preS1, preS2, and S regions. The large surface protein is translated from the first initiation codon of the 2.4-kb preS1 mRNA and contains preS1, preS2 and S sequences. The large surface protein is required for receptor binding for viral entry[19]. The middle surface protein is the product of the 2.1-kb preS2/S mRNA and contains preS2 and S sequences. The small surface protein is translated from both the 2.1-kb preS2/S mRNA and a slightly shorter S mRNA[20]. As mentioned above, the small surface protein is the most abundant surface protein in either SVPs or virions. The X gene ORF encodes the 17-kDa HBx protein, which is translated from the 0.7-kb X mRNA. HBx plays a regulatory role in the HBV lifecycle. It has multiple functions and is required for efficient viral replication *in* vivo[21].

## HBV lifecycle

### HBV entry into hepatocytes

HBV is a hepatotropic virus. Its entry into hepatocytes is mediated by its surface proteins, and the preS1 domain of the large surface protein plays a critical role [19,22]. The filamentous HBsAg subviral particles, which have a significant level of the large surface protein bind specifically to hepatocellular membranes, whereas spherical HBsAg subviral particles, which have a low level of the large surface protein, bind to the membranes to a smaller extent. The importance of the preS1 domain in mediating this membrane binding was confirmed by the observations that the anti-preS1 antibody and preS1-derived peptides could inhibit this binding [22–25], and by deletion-mapping analysis of the preS1 region of the large surface protein[19]. The middle surface protein, in contrast, is not necessary for the formation of the viral envelope[24]. The antigenic loop of the S protein contains an infectivity determinant as well. The deletions or mutations of this loop affected the infectivity of hepatitis delta virus (HDV), a defective virus that is coated by HBV envelope proteins[26].

Possible cellular and serum proteins that may serve as HBV receptors have been studied since the 1980s. Heparan sulfate proteoglycans, which are present on the cell surface, were found to promote the initial attachment of HBV to hepatocytes via the low-affinity binding to the antigenic loop of the S protein to facilitate the entry process[27]. The sodium taurocholate cotransporting polypeptide (NTCP, also known as SLC10A1) is identified as the HBV receptor[28]. NTCP is expressed specifically on hepatocytes and required for the uptake of bile salts into hepatocytes[29]. Its silencing suppresses HBV infection[28] while its expression in HepG2 cells, a human hepatoblastoma cell line that is not susceptible to HBV infection, allowed the cells to be infected by HBV [30–34]. The requirement of NTCP for HBV to initiate infection partially explains the liver tropism of HBV. There are controversies regarding whether the expression of human NTCP (hNTCP) in murine liver-derived cells could render those cells susceptible to HBV infection [34,35]. In any case, the expression of hNTCP in the liver of mice allowed these mice to be infected by HDV but not HBV [36,37]. Since the introduction of HBV genomic DNA into mouse hepatocytes such as in HBV transgenic mice or via hydrodynamic injection led to HBV gene expression and viral replication in mouse hepatocytes [38,39], it is possible that additional host factor(s) in the early stage of HBV infection, such as at the step of viral entry or genomic DNA repair, are required for HBV to efficiently initiate a successful infection *in vivo*. The possibility that a restriction factor is present in mouse hepatocytes to prevent the initiation of HBV infection appears unlikely, as heterokaryons, which were generated by fusing hNTCP-expressing mouse liver-derived cells to HepG2 cells, were permissive to HBV infection[40]. The epidermal growth factor receptor (EGFR) is an additional factor that was also found to be involved in HBV entry mediated by NTCP[41].

Whether the internalization of HBV is dependent on caveola or clathrin is still a subject of debates. Caveolin-1 is a structural component of caveolae, which are plasma membrane microdomains enriched in cholesterol and sphingolipids. One the one hand, it was shown that the cellular uptake of HBV was dependent on the caveola-mediated endocytosis pathway in the neutral pH, bypassing the acidic endosomal compartments of the clathrin-mediated pathway[42]. This entry pathway was supported by the observation that ammonium chloride or bafilomycin A1, which inhibits the acidification of endosomes and lysosomes, had no effect on HBV infection of HepaRG cells, a hepatoma cell line that is susceptible to HBV infection upon the induction of their differentiation[43]. On the other hand, the internalization of HBV into HepG2 cells that stably expressed NTCP (HepG2-NTCP) was found to be dependent on the clathrin-mediated pathway and the actin cytoskeleton, as the inhibition of the clathrin-mediated endocytosis suppressed the internalization of HBV [44,45].

### Nuclear transport and repair of HBV genomic DNA

How the HBV core particle is released from internalized membrane vesicles into the cytosol is unclear, although fusogenic sequences located in the N-terminus of the preS1 protein and the N-terminus of the S protein that may mediate the fusion between the viral envelope and cellular membranes for the release of the core particle had been proposed [46,47]. After the release of the core particle into the cytosol, the core particle is transported to the nucleus. The studies using duck hepatitis B virus (DHBV) as a model indicated that this cytoplasmic trafficking relied on microtubules and their dynamic turnover[48]. This dependence on microtubules for trafficking was subsequently also observed for HBV core particles[49]. The core particles enter the nucleus via the nuclear pore complexes (NPC) without the need of disassembly, as the NPC allows the transport of particles up to 39 nm in diameter[50]. The binding of the core particle to NPC requires the phosphorylation of the core protein, which causes the exposure of the nuclear localization signal (NLS) located in the C terminal domain (CTD) of the core protein[51]. The exposed NLS can interact with the transport receptor importin α/β for the entry of the core particle into the nuclear basket [50,52], where the core particle binds to nucleoporin 153, a component of the NPC[53]. At that stage, the core particle disintegrates and releases the genomic DNA into the nucleoplasm.

For the partially double-stranded HBV genomic DNA to serve as the template for viral RNA synthesis, it must first be converted to the covalently closed circular DNA (cccDNA)[54]. The 5’-end of the minus strand of the HBV rcDNA is covalently linked to the polymerase. This protein must be removed before the rcDNA can be converted to the cccDNA. The removal of the terminal protein apparently occurs in the cytoplasm, as the protein-free rcDNA (PF-rcDNA) was detected in the cytoplasm of stable HepG2-NTCP cells that had been infected by HBV at 12 hours post-infection, much earlier than the appearance of the cccDNA in the nucleus[55]. The deproteinization of rcDNA likely takes place in the core particle or a unique subcellular compartment, as PF-rcDNA is insensitive to the ectopically expressed cytoplasmic exonuclease TREX1 during *de novo* HBV infection[56].

After the removal of the terminal protein, additional steps must be completed before the cccDNA can be generated. These steps include the removal of one copy of the 5’ terminal redundant segment (r) on the minus strand, the removal of the 5’-capped RNA fragment from the plus strand, the extension of the plus strand for completion, and the ligation of both DNA strands to seal the gap. The extension of the plus-strand may be completed either by the HBV DNA polymerase or by the host DNA repair mechanism. Studies using DHBV as a model support the involvement of the host repair mechanism, as the inhibitors of viral DNA polymerase failed to block the formation of cccDNA after viral infection, although they inhibited viral DNA replication[57].

The conversion of the rcDNA to the cccDNA involves a number of enzymes, which include a tyrosyl-DNA phosphodiesterase 2 (TDP2) to remove the terminal protein linked to the 5’-end of the minus strand[58], an endonuclease to remove the capped RNA primer at the 5’-end of the plus strand, the DNA polymerase κ and α to complete the synthesis of the fully double-stranded DNA [59,60], and DNA ligases 1 and 3 to join the 5’- and 3’-ends of both DNA strands[61]. In addition, the involvement of DNA topoisomerases for cccDNA synthesis had also been demonstrated[62]. More recently, an elegant study using yeast extracts, which support the conversion of rcDNA to cccDNA, was conducted to identify protein factors that may be involved in the synthesis of the HBV cccDNA. In the screening of yeast extracts, five key components that are required for the formation of the cccDNA were identified. These five components, which are defined as the minimal set of enzymes that are required for the conversion of rcDNA to cccDNA, are proliferating cell nuclear antigen (PCNA), the replication factor C complex (RFC), DNA polymerase δ (POLδ), flap endonuclease 1 (FEN1) and DNA ligase 1 (LIG1)[63]. The human homologs of these proteins were next purified and shown to be capable of repairing recombinant rcDNA *in vitro*[63]. Among these factors, FEN1 and LIG1 are required for the repair of the minus strand[64]. The structure of the rcDNA resembles damaged cellular DNA. Thus, besides these five factors, additional protein factors such as ataxia telangiectasia mutated (ATM), a serine/threonine kinase that is activated by DNA double-strand breaks[65], had also been reported to be involved in the formation of HBV cccDNA. The topic on the repair of the HBV rcDNA has recently been reviewed, and readers are referred to this recent article for further details[66].

After the formation of cccDNA, nucleosomes bind to cccDNA to form a mini-chromosome [67,68], which is also associated with non-histone proteins as well as the HBV core protein[69] and HBx[70]. HBx is suspected to regulate the post-translational modifications of histones, including acetylation and methylation [70,71]. Without HBx, histones are methylated but not acetylated, resulting in low transcriptional activities of cccDNA [70–72]. This issue will be further discussed later.

### HBV RNA transcription

The HBV genome contains four promoters, which are the core promoter, the preS1 promoter, the S promoter, and the X promoter. The activities of these four promoters are further controlled by the two enhancer elements EN1 and EN2. EN1 and EN2 are positioned approximately 600 base pairs (bp) apart within the P ORF and the X ORF, respectively. All of the HBV RNA transcripts terminate at the same polyadenylation site located in the C ORF[73]. A posttranslational *cis* regulatory element (PRE), which overlaps with a part of the X ORF, promotes the nuclear export of the unspliced HBV RNAs to the cytoplasm[74]. The locations of these regulatory elements in the HBV genome are shown in Figure 1. In this section, we will first discuss individual HBV promoters and enhancers followed by the discussion of transcription factors (TFs) that participate in viral RNA transcription. We will finally discuss the coordination of HBV transcription units and the post-transcriptional modification of HBV RNAs.

#### The core promoter

The core promoter drives the transcription of the precore protein RNA (pcRNA) and the core protein RNA (i.e., pgRNA) from two initiation sites that are located 30 nucleotides apart. The core promoter comprises the basal core promoter (BCP) and the upstream regulatory region (URR)[75]. The BCP alone is sufficient to direct the transcription of pcRNA and pgRNA[76]. This promoter lacks the canonical TATA-box [76,77], which is recognized by TATA-binding protein (TBP) for the selection of transcription initiation site. The URR regulates the BCP activity. It is composed of the core upstream regulatory sequence (CURS) and a negative regulatory element (NRE). CURS is at the proximal end of the BCP while NRE is at the distal end. The CURS enhances the BCP activity to produce pcRNA and pgRNA in a position- and orientation-dependent manner[76]. In contrast, the NRE suppresses the core promoter activity in a position-dependent but orientation-independent manner[78]. The NRE can be further divided into three subregions NRE-α, NRE-β, and NRE-γ. These three regions individually have weak suppressive activity on the core promoter but together they generate a strong synergistic suppressive effect[79].

#### PreS1 and S promoters

The preS1 promoter drives the transcription of the 2.4-kb preS1 mRNA. Unlike the core promoter, the preS1 promoter contains a TATA-box and has a unique transcription initiation site. In contrast, the S promoter, which does not have a TATA-box, drives the transcription of 2.1-kb preS2/S mRNA with multiple transcription initiation sites that spread over a length of ~35 nucleotides [20,80]. The RNA initiating upstream of the translation start codon of the preS2 protein can direct the synthesis of both the preS2 protein and the S protein, and those initiating downstream of it can only direct the synthesis of the S protein[20].

#### The X promoter

The X promoter directs the transcription of the 0.7-kb X mRNA, although a 3.9-kb X mRNA that was the result of bypassing the polyadenylation site once had also been detected in cell culture studies [81,82]. This 3.9-kb X mRNA contains two copies of the HBx coding sequence. Although it also contains two copies of the PRE, its nuclear export is inefficient[82]. The biological significance of this 3.9-kb X mRNA remains to be determined.

#### The enhancers

The HBV enhancers EN1 and EN2 overlap with the X promoter[83] and the core promoter[84], respectively. EN1 and EN2 control the activities of all four HBV promoters. For instance, the core promoter requires EN1 and EN2 for the enhancement of its activity[85]. EN1, with a length of nearly 300-bp for its optimal activity, substantially increases the activities of the core promoter and the X promoter, and has a more marginal effect on preS1 and S promoters[86]. EN1 possesses three domains, a 5’ modulator, an enhancer core, and a 3’ region overlapping the X promoter [83,87]. The central enhancer core is sufficient to provide the enhancer function and is referred to as the primary functional region of EN1^90^. Although the 5’ modulator does not have enhancer activity, it plays an accessory role to enhance the activity of the core domain. EN2 stimulates mainly preS1, S and X promoters.

#### The transcription factors

All of the transcriptional regulatory elements in the HBV genome harbors binding sites for hepatocyte-enriched TFs, which also contribute to the hepatotropism of HBV (Table 1). For instances, hepatocyte nuclear factors (HNF) 1 and 3 are required to activate the preS1 promoter [88,89] and EN1 and EN2 enhancers [90–94]. Similarly, the CCAAT-enhancer binding protein (C/EBP), which is also enriched in hepatocytes, binds to the S promoter [95,96], the preS1 promoter, the core promoter[97], and the EN2 enhancer[85]. C/EBP positively regulates the S promoter activity and, in the meantime, negatively regulates the preS1 promoter activity. It thus plays an important role in maintaining a low level of the preS1 RNA transcript[96].

**Table 1.** Hepatocyte-enriched transcription factors that regulate HBV promoters and enhancers

Transcription factors	Regulatory elements	References
HNF1 and HNF3	PreS1 promoter	91, 92
	EN1 and EN2	93–97
HNF4	Core promoter	102, 103
C/EBP	S promoter	98, 99
	PreS1 promoter	99
	Core promoter	100
	EN2	99
KLF5	S promoter	101
	Core promoter	101

Another liver-enriched TF Klf15 binds to the S promoter and the core promoter to activate these two promoters[98], and HNF4, a hepatocyte-enriched nuclear receptor, also binds to and activates the core promoter [99,100]. The activity of HNF4 on the core promoter is inhibited by the nuclear orphan receptor testicular receptor 4 (TR4)[101] as well as by the NRE that is located in the upstream region of the HNF4 binding site[79]. The NRE antagonizes the HNF4 activity in HeLa cervical carcinoma cells but not in Huh7 hepatoma cells due to the binding by multiple protein factors to NRE-α and NRE-β in Huh7 cells [79,100]. Thus, the NRE also contributes to the hepatotropism of the core promoter[102]. Hepatocyte-enriched transcription factors that are known to regulate HBV gene expressions are listed in Table I.

The HBV gene transcription is also regulated by ubiquitous TFs. Sp1 binding sites are found in ENII and the core promoter and play a dual role in HBV gene expression. The Sp1 binding site in ENII positively regulates core, S and X promoters. Its two binding sites in the core promoter also positively regulate the core promoter. However, the upstream Sp1 binding site in the core promoter also negatively regulates S and X promoters[103]. In addition to HNF4 and TR4, other nuclear receptors including retinoid X receptor α (RXRα), peroxisome proliferator-activated receptor α (PPARα) [104–106], farnesoid X receptor (FXR)[107], chicken ovalbumin upstream promoter TFs (COUP-TFs) [106,108], and TR2^112^ also regulate the activities of the core promoter and ENI and ENII enhancers. Two androgen response elements (AREs) that are recognized by the androgen receptor (AR) are also located in the immediate upstream region of the EN1 enhancer [109,110]. A DNA repair modulator, poly(ADP-ribose) polymerase 1 (PARP1), had also been shown to bind to an octamer motif in the core promoter to activate the core promoter[111]. The transcription factors RFX1 and MIBP1 had also been shown to bind to the NRE-γ motif of the NRE[112]. More detailed descriptions of TFs that regulate HBV gene expression are discussed in other review articles [113–117].

#### The coordination of HBV transcription units

As the four transcription units in the HBV genome overlaps with one another extensively, how these transcription units are coordinated to transcribe HBV RNAs is unclear. A temporal regulation of HBV gene expression had been proposed, with the X gene being the early gene [118,119]. The early expression of the X gene will also be consistent with the observation that HBx is required to enhance HBV RNA transcription, which will be discussed later [92,120]. This temporal regulation of gene expression will likely involve the activities of various *cis*- and *trans*-acting factors that positively and negatively regulate HBV gene expression. For examples, COUP-TF1 and TR4 may suppress the core promoter in the early stage of viral infection to allow the expression of X and other HBV genes [101,121].

#### Post-transcriptional modifications of HBV RNAs

HBV RNAs can also undergo post-transcriptional modifications such as the N6-methyladenosine (m^6^A) modification. The m^6^A modification sites have been identified and are in the conserved epsilon (ε) stem-loop structure, which is present at both ends of the pgRNA. The m^6^A site in the 5’ ε structure is required for efficient reverse transcription of the pgRNA and the m^6^A modification in the 3’ ε structure destabilizes all HBV RNA transcripts[122]. Two cellular proteins YTHDC1 and FMRP bind to m^6^A and facilitate the nuclear export of the HBV RNA transcripts. Thus, the m^6^A modification of the HBV RNAs plays an important role in the HBV lifecycle[123]. HBV pgRNA can also undergo alternative splicing to generate spliced RNAs. The topic of RNA splicing will be discussed later.

Several nuclear proteins can bind to HBV RNAs to promote their degradation and suppress viral transcription and replication. These proteins include a zinc-finger antiviral protein (ZAP)[124], an RNA helicase (SKIV2L), which binds to the X mRNA [125], a ribonuclease (ISG20), which recognizes the ε structure, the packaging signal in the pgRNA [126,127]. These studies had been reviewed elsewhere[128], and will not be repeated here.

### HBV protein translation

The translation of HBV mRNAs is mediated by the cap-dependent manner, with the exception of the polymerase, for which the mechanism is still not fully understood. The polymerase is translated from the pgRNA, which contains the core protein coding sequence at the 5’-end and the polymerase coding sequence partially overlapping the core protein sequence in a different reading frame at the 3’-end. As core and polymerase proteins accumulate in hepatocytes at a ratio of 200–300 to 1, the translation for the polymerase is significantly less efficient than that for the core protein. Two models have been proposed to explain how the polymerase may be translated. The first model is a leaky scanning mechanism, in which a small proportion of ribosomes landed at the 5’-end of the pgRNA bypass the core initiation codon and peruse the mRNA until they arrive at the polymerase AUG codon [129,130]. The presence of multiple AUG codons between the core AUG codon and the polymerase AUG codon would appear to argue against the leaky scanning mechanism. The second model, which was based on the studies of DHBV, suggests that the polymerase is translated via a ribosome shunting mechanism. In this model, ribosomes landed near the 5’-end of the pgRNA are shunted from the 5’-end to an acceptor site located at or near the polymerase AUG codon [131,132]. In spite of its structural similarity to pgRNA, the pcRNA is not used for the synthesis of the polymerase[17], indicating that the translation of the precore sequence suppresses the translation of the downstream polymerase sequence.

### HBV proteins

#### The core protein

The core protein is a 21-kDa protein with an arginine-rich, protamine-like C-terminal domain (CTD) that is required for pgRNA packaging. Its N-terminal 1–149 residues form an assembly domain and are highly α-helical in structure. The assembly domains of two monomers form a T-shaped dimer, with the stem region constituting the dimer interphase and the arm tips making the polymerization contacts. The stem protrudes from the capsid surface like a spike[133]. An HBV nucleocapsid is assembled by 90 or 120 dimers of the core protein[9].

The assembly domain contains a 9-amino acid (aa) linker (residues 141–149) at the C terminus, which is connected to the CTD. The CTD has three major phosphorylation sites and several additional serine and threonine residues that may be used for phosphorylation [134,135]. The phosphorylation of the CTD is required for pgRNA packaging and its dephosphorylation, which apparently takes place during pgRNA packaging[135], is required for viral DNA replication [136,137]. It was first reported in 1980 that Dane particles contained an endogenous kinase activity[8]. The candidate kinases that had been suggested include cyclin-dependent kinase 2 (CDK2) [138,139], polo-like kinase (PLK)[140], protein kinase A (PKA)[141], protein kinase C (PKC)[142], and serine/arginine-rich protein-specific kinases (SRPKs) [143,144]. Two different phosphatases protein phosphatase 1 (PP1) and protein phosphatase 2A (PP2A) had also been identified as the possible phosphatases that regulate the dephosphorylation of the core protein [145,146]. The arginine-rich sequence of the core protein also contains a NLS that is important for the nuclear import of the core protein/particle[147], and a nuclear export signal (NES) that binds to Tip-associated protein/nuclear export factor-1 (TAP/NXF1)[148]. The possession of both NLS and NES allows the core protein to shuttle between cytoplasm and the nucleus[148].

#### The precore protein and HBeAg

The C gene ORF contains two in-phase ATG codons separated by 28 codons termed the precore region. The translation of the core protein is initiated from the downstream AUG codon and the translation initiating from the upstream AUG codon produces the precore protein (p25). The precore protein contains a signal peptide, which is constituted by its N-terminal 19 amino acids. This signal peptide guides the precore protein to the ER where it is removed by the signal peptidase to generate the precore protein derivative p22. p22 is further cleaved at the C-terminal arginine-rich domain by a furin-like protease in *trans*-Golgi to generate the 17-kDa HBeAg and secreted[149]. Part of p22 is released back into the cytosol and, due to the presence of the NLS in its C-terminus, can also be translocated into the nucleus[150]. The cytosolic p22 may also be phosphorylated[151]. Although p22 contains the entire sequence of the core protein plus an amino-terminal extension of 10 amino acids, it cannot form the core particle due to the presence of a cysteine residue in the precore sequence and can act as a dominant negative factor to suppress the formation of the core particle[152]. Indeed, the over-expression of the precore protein has been shown to suppress HBV replication in a mouse model[153]. p22 has also been shown to inhibit the interferon signaling pathway by suppressing the nuclear import of STAT and the induction of interferon-stimulated genes (ISGs)[154]. HBV with mutations in the precore region such as the G to A mutation at nucleotide (nt) 1896, which converts a TGG codon to the TAG termination codon and abolishes the expression of the precore protein, are frequently detected in chronic HBV patients[155], indicating that the precore protein and hence HBeAg are not essential for HBV infection. However, studies indicate that secreted HBeAg have immunoregulatory functions. It can transiently suppress the innate immune response by suppressing the Toll-like receptor (TLR) signaling pathway [156–158]. In addition, by using mice as a model, it was found that maternal HBeAg could educate Kupffer cells, the resident macrophages of the liver, of the offspring, and when Kupffer cells of the offspring were exposed to HBeAg again, these Kupffer cells would undergo the M2 anti-inflammatory polarization to suppress HBV-specific T cell activities to result in HBV persistence[159]. This finding provides an explanation as to why children born to HBeAg-positive mothers would often become chronic carriers of HBV without intervention[160].

#### The surface antigens

The three envelope proteins of HBV, L, M and S surface proteins, are synthesized from the S ORF from three different in-phase initiation codons. As such, these three proteins are co-carboxy-terminal with different amino-terminal extensions. The S protein is 226-aa in length. The M protein contains the entire sequence of the S protein plus an extra 55-aa preS2 domain, whereas the L protein carries an additional preS1 domain that is 108 or 119 aa in length, depending on the genotypes. The S protein has four transmembrane (TM) segments embedded in the ER membrane, with both ends extruding into the ER lumen and thus containing one luminal and two cytosolic loops[161]. The N-terminal two TM segments contain two topogenic signals for their proper positioning in the lipid bilayer[162]. These three envelope proteins share an N-linked glycosylation site at asparagine-146 (Asn-146) in the luminal loop[163]. This glycosylation site is only partially used, resulting in the generation of both glycosylated and non-glycosylated forms of surface proteins[164]. This glycosylation site also contains the HBsAg antigenic epitope exposed on the surface of the viral envelope [165,166].

The preS2 domain of the M protein harbors a second N-linked glycosylation site at Asn-4. The N-linked glycan at this site, which interacts with the ER chaperone protein calnexin[167], is required for the efficient secretion of the virion and the M protein-containing SVPs [168,169]. In contrast, the glycosylation at Asn-146 shared by all three envelope proteins is not required for the secretion of the virion and SVPs[167]. It should be noted that, although the L protein also contains the preS2 domain, Asn-4 is not glycosylated in the L protein[164], probably due to its dual topology, which will be discussed below. In addition to the N-linked glycosylation, an O-linked glycosylation is also detected at threonine-37 (Thr-37) of the preS2 domain in a HBV genotype-dependent manner [167,170,171]. The role of the M protein in the HBV life cycle is still unclear, as the M protein is not required for viral replication, morphogenesis, and secretion in chronic HBV patients carrying HBV mutants incapable of expressing the M protein[172]. Furthermore, the M protein had also been shown to be dispensable for HBV assembly and infectivity in cell cultures[173]. Several studies, however, revealed a possible role of the M protein in hepatocarcinogenesis [174–177].

The L protein has two different topological configurations in the membranes. The N-terminal preS (i.e., preS1 and preS2) region of half of the L protein is localized to the cytosol and the other half is localized in the ER lumen [178,179]. The cytosolic localization of the preS region is the initial configuration after translation. In this case, the first TM domain (TM1) in the S sequence is not embedded in the ER membrane, resulting in the cytosolic localization of the preS region. This configuration is essential for the L protein to interact with the capsid particle for virion assembly. A short 21-aa fragment in preS1 and a portion of the cytosolic loop of the S sequence contribute to this capsid interaction [180–182]. The localization of the preS region in the ER lumen is a post-translational event. In this case, TM1 in the S sequence is embedded in the membrane. The exposure of the preS sequence on the surface of the viral particle is essential for viral attachment [179,183,184]. The translocation of the preS region across the membrane requires the interaction of the L protein with the cytosolic chaperones heat shock cognate 70 (Hsc70), heat shock protein 40 (Hsp40)[185] and the ER luminal chaperone GRP78/BiP [186,187]. The C-terminus of the preS1 domain is critical for its post-translational translocation, as the deletion of aa. 70–94 results in the co-translational translocation of the preS domain into the ER lumen. This region has been named the cytosolic anchorage determinant (CAD) for its ability to interact with Hsc70 and suppress the co-translational translocation of the preS domain[188].

The first 77 amino acids of the preS1 region is vital for HBV infection based on the mutagenesis studies[19]. Gly-2 of preS1 is myristoylated and essential for HBV infection [189,190], and a synthetic myristoylated peptide containing Gly-2 and its downstream 46 amino acids of the preS1 sequence can efficiently inhibit HBV infection[24]. This 47-aa sequence is also required for HBV to interact with its receptor NTCP to initiate infection[28]. The replacement of the C14-myristate group in the preS1 peptide with various moieties of different hydrocarbon chain lengths, such as C5-pentanoyl, C8-octanoyl and C16-palmitoyl, revealed that a longer hydrocarbon chain length led to a better suppressive effect on HBV infection[24]. These results indicate that the acyl moiety likely participates in the binding to the cell surface receptor.

After the envelope proteins are integrated into the membrane, the cysteine residue in the S domain forms an intermolecular disulfide bond for the formation of a homodimer or a heterodimer to stabilize the virion or the SVP structure [191,192]. Both S and M proteins carry export signals for independent secretion, while the L protein is retained in the cytoplasm in the absence of S and M proteins. As such, the secretion of the S protein is repressed when it is co-expressed with a high level of the L protein [193,194].

Besides being a structural protein, L and M proteins can also function as transcriptional transactivators for host genes as well[195]. L and M mutants with C-terminal truncations, which can be generated after the integration of HBV DNA into host chromosomes, have been detected in HBV-related HCC and can function as a transcriptional transactivator [195,196]. Their target genes include the growth factor α and NF-κB genes, and they may be involved in hepatocarcinogenesis [197–199]. Natural L protein mutants had also been identified in chronically infected patients[200]. Deletions in the preS1 or preS2 sequence can lead to the retention of the L protein in the ER and the induction of ER stress, which is seen in chronic HBV patients with or without HCC [201–203]. The envelope protein mutants may be selected due to their ability to promote the survival and proliferation of infected cells.

#### The DNA polymerase

The HBV DNA polymerase is a 90-kDa protein composed of four distinct domains, the terminal protein (TP) domain at the N-terminus, followed by a spacer region, the RT domain, and the RNase H domain at the C-terminus. Most of the currently approved drugs for the treatment of HBV infection are nucleotide or nucleoside analogs (NAs). They target the RT. TP serves as the primer for the minus-strand DNA synthesis by first binding to the ε stem-loop structure, which is the packaging signal located at the 5’ end of the pgRNA, forming a ribonucleoprotein (RNP) complex to initiate the encapsidation of the pgRNA[204]. A tyrosine residue in TP is covalently linked to the first nucleotide of the minus strand via a phosphodiester bond[205]. The binding of a single polymerase to the ε structure to initiate encapsidation indicates that the polymerase and the nucleocapsid likely exist in nearly equal amounts. This is supported by the observation that there is ~0.7 polymerase molecule per DNA molecule[204]. After the encapsidation of the pgRNA, the synthesis of the HBV DNA genome ensues, which takes place in the capsid. This will be discussed later.

#### HBx

The HBV X protein, or HBx, encoded by the X gene, is a 154-aa regulatory protein with a molecular weight of approximately 17.5 kDa. The first reported activity of HBx was its gene transactivation activity, which was found to activate a heterologous promoter[206]. Subsequent studies demonstrated a critical role of HBx in activating HBV gene expression and replication both *in vitro* and *in vivo* [120,207,208]. These gene transactivation activities of HBX will be discussed below. HBx is detected in both the nucleus and the cytoplasm. In addition to activating gene expression, it can also regulate signaling pathways[209]. HBx does not directly bind to DNA, but it can bind to selected TFs, especially TFs of the bZip family such as AP-1, AP-2 and ATFs, to stimulate their DNA binding activities and indirectly activate gene expression [210–212]. HBx also colocalizes with mitochondria and can affect mitochondrial physiology and promote mitophagy, the selective removal of mitochondria by autophagy [213–215].

**Figure 2b.**
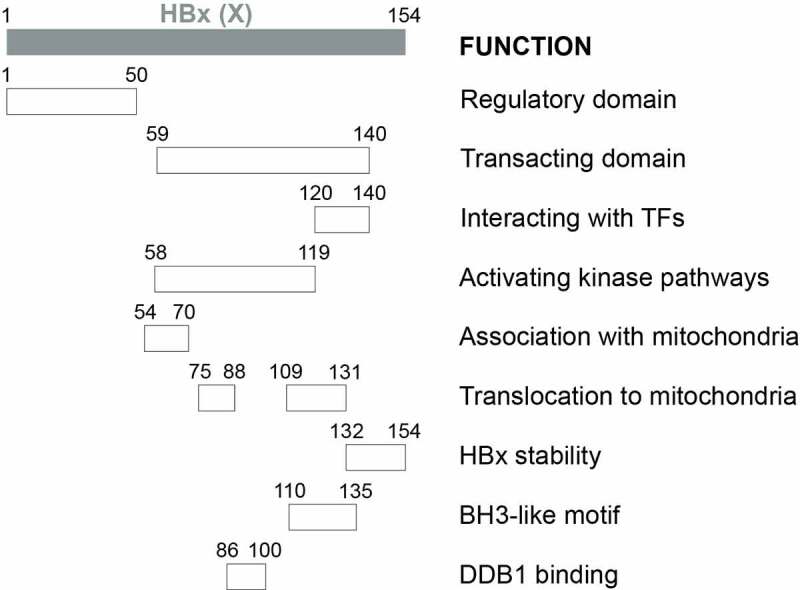
Continued.

Results from deletion analysis indicated that HBx proteins could be divided into two major functional domains (Figure 2a). The first domain located at the N-terminal one-third (residues 1–50) is a self-inhibitory domain (i.e., the regulatory domain) that prevents excessive transactivation[216]. The second domain located at the C-terminal two-thirds (residues 59–140) is responsible for transactivation functions (i.e., the transacting domain)[217]. While residues 120–140 mediate the interaction with TFs in the nucleus to activate gene expression [217,218], residues 58–119 are important for activating kinase pathways[219]. The studies on the localization of HBx to mitochondria revealed additional activities of the transacting domain, with residues 54–70 required for the association with mitochondria and residues 75–88 and 109–131 aiding the translocation of HBx to mitochondria[220]. The 23 aa at the C-terminal is required for the HBx stability and functions[221]. Note that the above findings were derived from deletion and mutagenesis analyses. The possibility remains that these mutations may alter the structure of HBx and affect the study results. The determination of the crystal structure of HBx will help to resolve this concern[92]. In this regard, two recent reports, which studied the crystal structure of the BH3-like motif (residues 110–135) of HBx in association with the anti-apoptosis protein Bcl-2 or Bcl-xL, revealed that this motif adopted an amphipathic α-helix structure and bound to the BH3-binding pocket of these two proteins [222,223]. An earlier study using a cell-free system also suggested that HBx could potentially dimerize[224].**Figure 2a.**
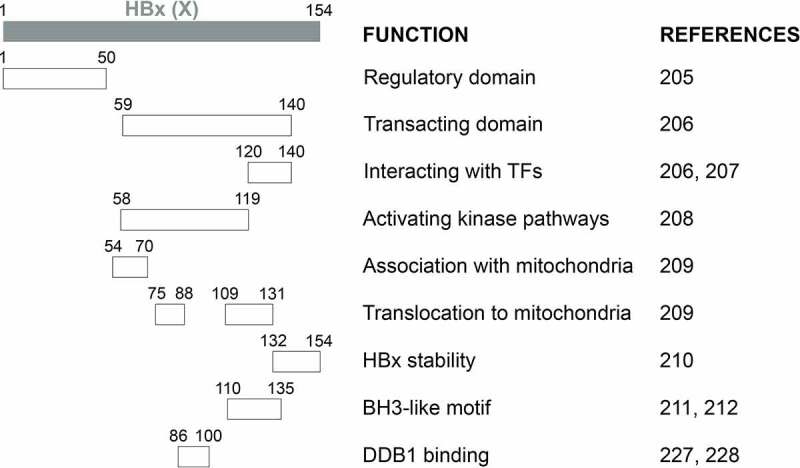
**Schematic illustration of HBx functional domains**. The functional domains of HBx that had been reported are shown.

##### Transactivation of HBV gene expression by HBx

By using transgenic mice that carried the wild-type HBV genome, the HBV genome that was incapable of expressing only HBx, and the HBV X gene, it was demonstrated that HBx could promote HBV gene expression to enhance viral replication[120]. This finding was confirmed in a separate mouse study, which employed hydrodynamic injection to introduce HBV genomic into mouse hepatocytes[208], and in a cell culture study, which demonstrated that the gene transactivation function of HBx was important for the augmentation of HBV replication[207]. The role of HBx in promoting viral RNA transcription and replication has been confirmed in many other studies [21,225–228], and had been reviewed in detail elsewhere[92].

The effect of HBx on viral RNA transcription and replication was shown to be dependent on damaged DNA binding protein 1 (DDB1)[229], as DDB1 binding-deficient HBx could not fully restore the replication of HBx-deficient HBV[230]. Curiously, in a separate study, it was found that HBx defective in binding to DDB1 could still fully support HBV replication and that the effect of DDB1 on viral RNA transcription is independent of HBx[231]. The reason for this discrepancy is unclear and might be related to the use of different cell culture systems in those studies.

By using a cccDNA-specific chromatin immunoprecipitation (ChIP)-based quantitative assay, it was found that HBx was recruited to the HBV minichromosome to prevent cccDNA deacetylation, verifying its role in the epigenetic regulation of cccDNA function[70]. In the absence of HBx, the level of the histone deactylases and hypoacetylated histones increased[70]. In addition, HBx interacts with epigenetic regulators, such as protein arginine methyltransferase 1 (PRMT1)[72]. PRMT1 suppresses HBV gene expression, and this suppression is dependent on its methyltransferase activity. The binding of HBx to PRMT1 inhibits the protein methylation activity of PRMT1 and relieves this suppression to enhance HBV gene expression[72]. Moreover, HBx induces DDB1 to degrade WD repeat domain 77 protein (WDR77), which enhances the methyltransferase activity and represses HBV replication[232]. A recent study also showed that HBx recruited m^6^A methyltransferase complexes to promote the co-transcriptional m^6^A modification of viral RNAs, which was discussed in section 3.3.7 above[233].

##### Transactivation of host gene expression by HBx

Besides activating HBV viral replication and gene expression as described above, HBx can also transactivate host genes, such as the genes of class I major histocompatibility complex (MHC)[234], inducible nitric oxide synthase (iNOS) [235,236], and interleukin-8 (IL-8)[237]. (Please see the previous review for a more complete list[238].) These activities of HBx are mediated by its binding to a wide variety of TFs, including AP-1 and AP-2[210], ATF/CREB[211], C/EBP[239], E2F[240], NF-AT[241], p53 [242,243], HIF-1α[244], HNF1[245], SMAD4[246], STAT-3 and NF-κB[247], sterol regulatory element-binding protein (SREBP)[248], and many others. HBx has also been shown to bind to the androgen receptor (AR) to promote its nuclear localization in the presence of dihydrotestosterone and enhance its gene transactivation activity[249]. HBx can also serve as a transcriptional activator by associating with several basal transcriptional factors[250], including RNA polymerases subunit RPB5[251], TBP [252,253], TFIIB[254], and TFIIH[255].

The co-activators CREB-binding protein (CBP) and p300 interact with CREB to stimulate gene expression. HBx interacts with CBP/p300 both *in vitro* and *in vivo* to activate the expression of genes such as IL-8 and PCNA[256]. HBx also facilitates the CREB-mediated activation of miR-3188, a microRNA (miRNA) that is overexpressed in HBV-related HCC, suggesting a possible role of the HBx-CREB-miR-3188 pathway in hepatocarcinogenesis[257]. HBx-CREB also upregulates the centrosomal P4.1-associated protein (CPAP), and CPAP also interacts with HBx to enhance cell proliferation and migration. These findings suggest a role of the CPAP and HBx interaction in HBV-induced hepatocarcinogenesis[258].

##### Interplay between HBx and the ubiquitin-proteasome system

One of the HBx cellular targets is the proteasome complex, which was initially identified by the yeast two-hybrid screening study [259,260]. HBx was found to suppress the activity of the proteasome. A further study indicated that HBx could also be degraded by the ubiquitin-proteasome pathway and had a short half-life of 30 minutes[261]. This finding is similar to the finding of a previous report, which indicated that two-thirds of HBx were soluble and had a half-life of 15 minutes, and the remaining HBx that was associated with the nuclear framework had a half-life of 3 hours[262]. The treatment of cells with proteasome inhibitors led to the suppression of the gene transactivation activity of HBx, suggesting that the proteasome function is required for the transactivation activity of HBx. These findings revealed an intricate interplay between HBx and proteasomes.

Many studies have examined the interaction between HBx and the components of the ubiquitin-proteasome system (UPS), which include a E1 ubiquitin-activating enzyme, a E2 ubiquitin-conjugating enzyme, a E3 ubiquitin ligase, and the 26S proteasome. HBx can bind to the α7 subunit (PSMA7) of the 20S proteasomal core[259], and PSMC1, a regulatory subunit of the 26S proteasome[263]. These bindings are mediated by residues 132–139^265^, a sequence homologous to the Kunitz domain of Kunitz-type serine protease inhibitors[264]. These bindings are important for the gene transactivation function of HBx.

HBx can also interact with Cullin-RING ligase 4 (CRL4), a E3 ubiquitin ligase complex. This E3 complex consists of cullin protein 4 (CUL4), DDB1, and a RING (really interesting new gene) protein. DDB1 binds a subset of DDB1 cullin accessory factors (DCAFs) to recruit protein substrates for ubiquitination and degradation. UV-DDB, as a part of the E3 ubiquitin ligase complex, initiates the nucleotide excision repair (NER) by recognizing damaged chromatin and concomitantly ubiquitinating core histones at the lesion. HBx binds to DDB1 and interferes with the NER, resulting in the accumulation of DNA mutations in hepatocytes that may contribute to the development of hepatocellular carcinoma (HCC) [265–268]. Another subunit of UV-DDB, DDB2, competes against HBx for binding to DDB1 to antagonize the cell death induced by HBx[269]. The crystal structure analysis revealed that an α-helical motif at residues 86–100 of HBx could bind to the large pocket enclosed by the double β-propeller domains (BPA-BPC) of DDB1 [270,271]. The binding of HBx to DDB1 and its association with CRL4 E3 ligase suggest that HBx may serve as a viral DCAF to alter the substrate specificity of CRL4^274^. Recent studies indicated that CRL4 hijacked by HBx could target the structural maintenance of chromosomes (Smc) complexes 5 and 6 (Smc5/6), which bind to the HBV cccDNA to suppress HBV gene expression, for degradation [272,273].

In addition to DDB, several proteins involved in DNA repair also interact with HBx. Among them is the large multi-subunit transcription factor IIH (TFIIH), which functions as a helicase to unwind DNA for the initiation of the NER after a DNA lesion has been identified. HBx interferes with the activities of TFIIH by binding to its subunits ERCC3 and ERCC2, as well as p53, a regulator of NER [255,274]. HBx also transcriptionally suppresses the expression of two other subunits of TFIIH, XPB (p89) and XPD (p80), through the interaction with the Sp1 TF[275]. Collectively, the association of HBx with the DNA repair machinery induces DNA damage in infected cells[276], implicating a role of HBx in hepatocarcinogenesis (for further details, see the previous review[277]).

##### HBx and cellular signaling pathways

HBx had also been shown to activate the Ras-Raf-MAP kinase signaling pathway to activate TFs AP-1 and NF-κB [278,279]. The activation of this pathway was abolished if HBx was fused to a NLS and localized to the nucleus. As the fusion to the NLS did not affect the ability of HBx to activate the EN1 enhancer, HBx clearly possesses distinct activities in both the cytoplasm and the nucleus[209]. The Ras-Raf-MAP kinase pathway suppresses HBV replication. However, this suppression is HBx-independent[280].

The cellular protein kinase in the signal transduction pathway stimulated by HBx includes, in addition to extracellular signal-regulated kinases (ERK) (i.e., MAP kinases)[214], c-Jun N-terminal kinases (JNKs)[281], Janus kinase (Jak)/STAT[282], phosphatidylinositol 3-kinase (PI3K)[283], proline-rich tyrosine kinase-2 (Pyk2) and Src kinase[284]. As mentioned above, HBx activates NF-AT and Src, which are known to be a calcium-stimulated TF and an effector of Pyk2, respectively. Pyk2, a cytoplasmic calcium-activated kinase, is activated by elevated cytosolic Ca^2+^ released from ER or mitochondria. Activated Pyk2 is autophosphorylated and bind to the SH2 domain of Src via its phosphorylated tyrosine-402, thereby leading to Src activation to promote the Ras-Raf-MAP kinase cascade. HBx can increase cytosolic Ca^2+^ levels by stimulating calcium entry into cells to activate Pyk2^228^[285], Blocking store-operated calcium entry (SOCE) or the inhibition of Pyk2 or Ca^2+^ signaling blocks the HBV DNA replication [226,285].

**Figure 3.**
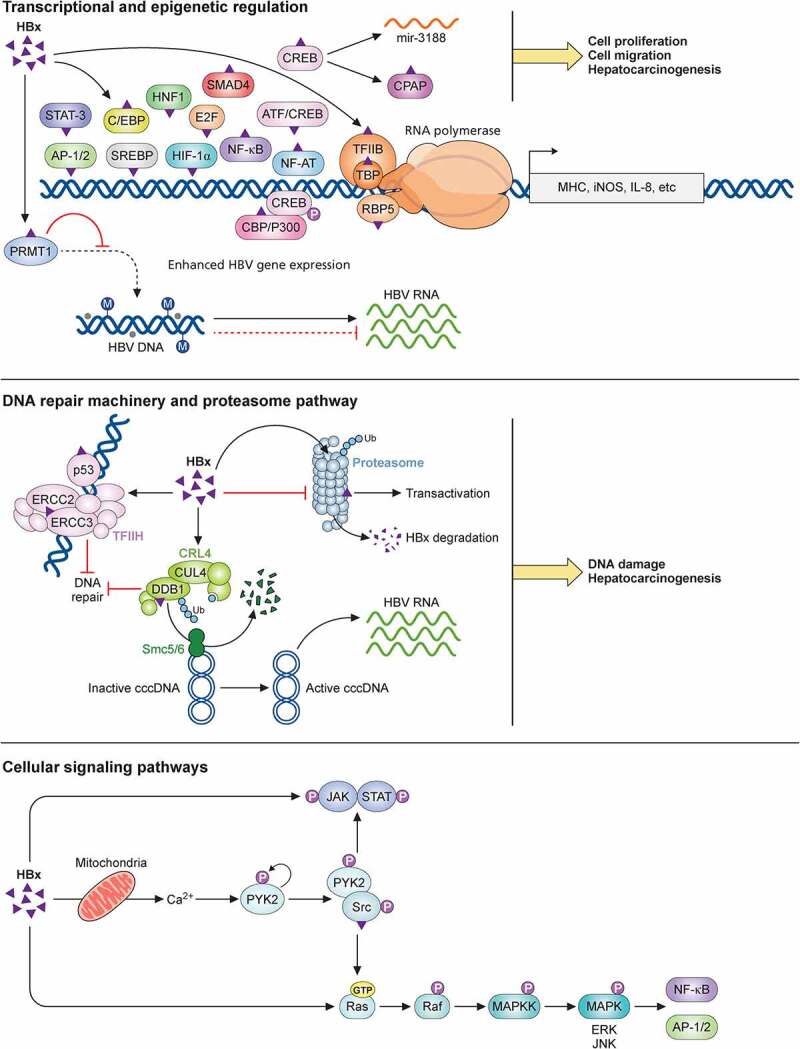
**Biological activities of HBx**. HBx regulates multiple cellular pathways. It can interact with many transcription factors to activate the expression of host genes, such as MHC class I, iNOS, and IL-8. Its regulation of the CREB-miR-3188 pathway may also play a role in the induction of hepatocarcinogenesis. HBx also epigenetically upregulates HBV gene expression by interacting with a methyltransferase, PRMT1. In addition, HBx suppresses the proteasomal function but is also degraded by the proteasome. It also interacts with the E3 ubiquitin ligase complex to promote the degradation of Smc5/6 and the activation of HBV gene expression. In addition, by interacting with the transcription factor TFIIH, HBx interferes with the host DNA repair machinery, resulting in the accumulation of host DNA damages. Finally, HBx can also activate cellular kinase signaling transduction pathways, including the interaction with mitochondria and the modulation of cytosolic Ca^2+^ levels to activate the Pyk2-Src pathway, and the activation of the Ras-Raf-MAPK pathway. The involvement of HBx in other pathways such as apoptosis, cell cycles, epigenetic signaling regulations, and autophagy is not illustrated in the figure.

In addition to the functions discussed above, HBx is also involved in cellular apoptosis resulting from its suppression of DNA repair and its interaction with p53[286], the regulation of cell cycles with different effects, epigenetic-signaling mechanisms, and autophagy (for details see other reviews [92,287,288]). In summary, HBx is a multifunctional regulator with diverse activities. Part of its biological activities is illustrated in Figure 3.

### HBV DNA replication

#### Packaging of pgRNA

The pgRNA, which is also the mRNA for the core protein and the polymerase, serves as the template for the replication of HBV DNA. Protein chaperones assist with the landing of the polymerase to the packaging signal ε at the 5’-end of pgRNA, forming the RNP complex and triggering the initiation of the encapsidation process.

The ε stem-loop structure contains a lower stem, an upper stem, a central bulge and an apical loop. It is located in the precore sequence, between the translation initiation codons of the precore protein and the core protein. Due to the terminal redundancy of the pgRNA, the ε structure is present at both ends of the pgRNA. However, only the 5’ ε structure participates in the viral DNA replication. Interestingly, the pcRNA, which also contains two ε structures, is not used for encapsidation. This is likely due to the translation of the precore protein and the disruption of the ε structure in the precore sequence by the 80S ribosomes during translation, thus preventing pcRNA from being packaged[289].

After the binding of the polymerase to the pgRNA, the core protein is recruited to encapsidate the RNP. The phosphorylation of the CTD domain of the core protein is essential for pgRNA packaging and its dephosphorylation is essential for the synthesis of the minus-strand DNA [137,290]. It was recently shown that the dephosphorylation of the core protein took place during the packaging of pgRNA[135]. This modification promotes reverse transcription and is beneficial to the stability of the nucleocapsids [290,291]. A recent report identified several sites in the pgRNA that interact with the CTD domain of the core protein to promote the nucleocapsid assembly[292].

Cellular proteins, such as the components of the Hsp90 chaperone complex, Hsp90, Hsp70, Hop, Hsp40, and p23, are involved in the encapsidation of the pgRNA [293–297], and chemical inhibitors that target these chaperon proteins strongly inhibit HBV DNA replication[297]. Chaperons may help with the folding and stabilize the structure of the polymerase to promote its binding to the ε structure[192]. Several nuclear proteins are involved in the pgRNA packaging as well. RNA-binding motif protein 24 (RBM24) mediates the interaction between the polymerase and the ε structure[298]. The eukaryotic translation initiation factor (eIF4E) is also recruited into the RNP complex[299]. A nucleophosmin B23 binds to the core protein dimer to promote the capsid assembly and suppress the dissociation of core proteins[300].

#### Minus-strand DNA synthesis

Following the packaging of pgRNA, the reverse transcription for the synthesis of the minus-strand DNA is initiated. This DNA synthesis takes place inside the nucleocapsid and involves several steps. The first step is the priming reaction, which uses the sequence 5’-UUC-3’ in the central bulge of the ε structure as the template and the polymerase TP domain as the primer. The tyrosine-63 residue in the polymerase TP domain is covalently linked to deoxyguanosine monophosphate (dGMP), which base-pairs with the 3’ C in the bulge[301]. The next step is the addition of two deoxyadenosine monophosphates (dAMPs) to generate the sequence 5’-GAA-3’. After these initial priming and elongation steps, this polymerase and oligomer complex is translocated to the 3’ end of the pgRNA, where it binds to the complementary direct repeat 1 (DR1) sequence. The 5’ ε structure and the acceptor site are likely located in the proximity of each other in the nucleocapsid to promote this template switch. A short *cis*-acting element phi (φ) located upstream of the acceptor site, which base-pairs with the 5’ half of the ε structure, is required for efficient minus-strand DNA synthesis[302].

The third step is the elongation of the minus strand to the 5’ end of the pgRNA template. The completion of this DNA synthesis will generate a short terminal redundancy in the minus strand. This terminal redundancy is referred to as “r” and is necessary for the plus-strand DNA synthesis. During the elongation of the minus strand, the pgRNA is degraded by the RNase H activity of the polymerase, leaving behind an 18-nt 5’-capped RNA fragment containing the 11-nt DR1 sequence.

An interesting discovery is the requirement of autophagy for the replication of HBV DNA. It was discovered that the knockout of ATG5, a gene essential for autophagy, abolished HBV DNA replication in the mouse liver with only a marginal effect on pgRNA packaging [303,304]. How autophagy regulates HBV DNA replication remains largely unclear.

#### Plus-strand DNA synthesis

The primer for the plus-strand DNA synthesis is the 18-nt 5’-capped RNA fragment derived from the pgRNA. This RNA fragment contains the DR1 sequence at its 3’-end. The first step of the plus-strand DNA synthesis is the translocation of the RNA primer to the DR2 sequence located near the 5’-end of the minus-strand DNA. DR2 has the same sequence as DR1. This is the second template switch. Several *cis*-acting sequence elements in the minus strand that may base-pair and help to juxtapose both ends of the minus strand to facilitate this second template switch have been identified [305,306]. When the synthesis of the plus-strand DNA reaches the 5’-end of the minus-strand DNA template, a third template switch is essential for the continuation of the DNA synthesis. The terminal redundancy at both ends of the minus strand will help with this switch, leading to genome circularization to generate the RC DNA. The completion of the encapsidation process and the subsequent envelopment may limit the access of the replicating DNA to cytoplasmic dNTP pool and thus generate the partially double-stranded genome with a plus strand that terminates at different locations of the minus strand. Indeed, the plus-strand DNA synthesis can be resumed *in vitro* if the envelope is removed by a non-ionic detergent and dNTPs are provided[307].

A small fraction of the RNA primer (5%-20%) may also initiate the synthesis of the plus-strand DNA *in situ* without the second template switch, resulting in the generation of the double-stranded linear DNA (dslDNA). The dslDNA cannot produce functional pgRNA, due to the truncation of the unique polyadenylation signal. However, it can integrate into host chromosomes at the sites of double-stranded DNA breaks [308,309].

#### Amplification of cccDNA

The replicated RC DNA in the nucleocapsid may be enveloped and released from infected hepatocytes or may re-enter the nucleus to amplify the cccDNA pool. Recent studies using HepG2-NTCP cells infected by HBV indicated that the average cccDNA copy number was about 5–12 copies per cells throughout a six-week study period, and by using a nucleoside analog to inhibit HBV DNA replication, the half-life of cccDNA was found to be about 40 days[310]. Both intracellular recycling of rcDNA and the secondary infection contribute to the stable cccDNA pool in infected cells. The study of the liver of a duck chronically infected by DHBV indicated that 90% of the nuclei of duck hepatocytes contained between 1–17 copies of cccDNA, with the remaining 10% of hepatocytes containing more, and that the copy numbers of cccDNA may fluctuate over time[311]. The level of cccDNA can also be regulated by viral envelope proteins. Among the three envelope proteins, the L surface protein plays a primary role. Its expression alone or together with M and S surface proteins results in the reduction of the levels of cccDNA, and the suppression of its expression leads to the increase of cccDNA [312,313]. The stability of cccDNA in infected hepatocytes is a major reason as to why chronic HBV infection is difficult to treat.

### Viral maturation and egress

The final step of HBV morphogenesis is the envelopment of the nucleocapsid. HBV nucleocapsids interact with the preS1 region of the L surface protein and aa 56–80 in the first cytosolic loop of the S surface protein[181]. Although earlier EM studies revealed the budding of HBV core particles into the ER lumen to form mature viral particles[314], more recent studies also indicated an important role of multivesicular bodies (MVBs) in the formation and release of mature HBV particles[315]. MVB-associated endosomal sorting complexes required for transport (ESCRT)-I, -II, and -III[316], and additional factors involved in the ESCRT pathway, including α-taxilin[317], Nedd4[318], Vps4 and γ2-adaptin[319], are required for HBV egress. The autophagic pathway had also been suggested to be involved in the envelopment of HBV capsid particles and the release of mature virions[320].

**Figure 4.**
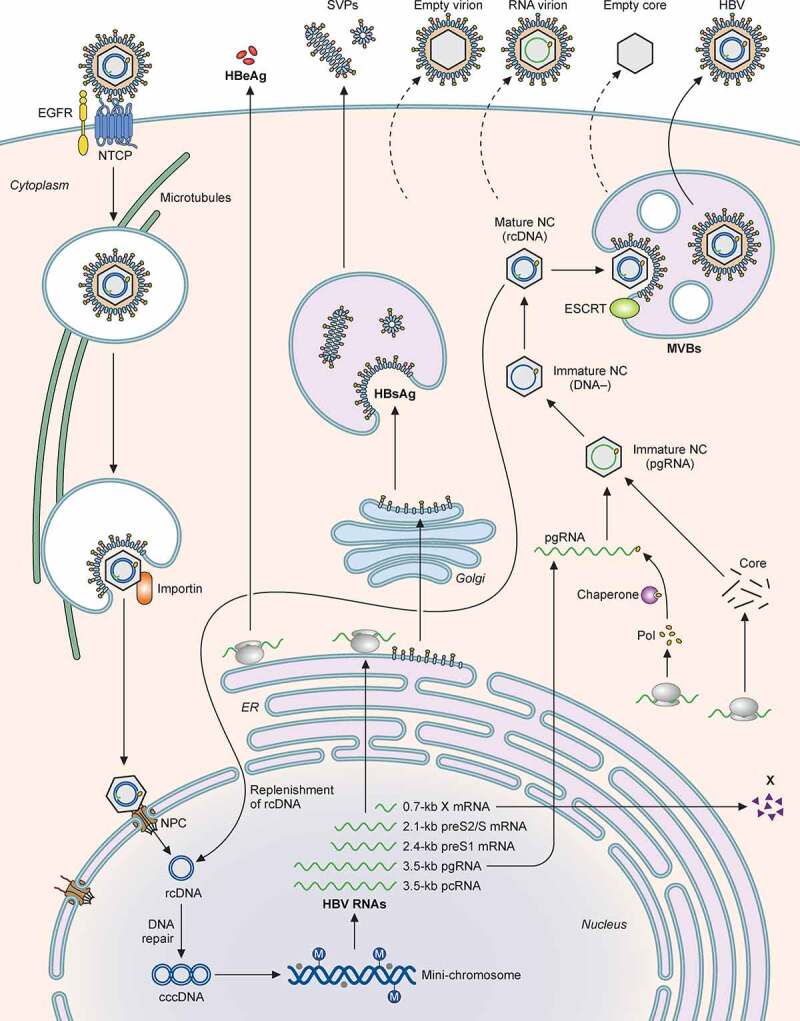
**Illustration of the HBV life cycle**. HBV initiates its infection of hepatocytes by binding to its receptor NTCP on the cell surface, with the assistance of EGFR. This results in the internalization of the viral particle via a pathway that is still unresolved. The nucleocapsid is subsequently released from the internalized membrane vesicles and transported to the nucleus in a process that involves microtubules. The nuclear transporter factor importin α/β then guides the capsid particle into the nuclear basket, where the HBV genome is released to the nucleoplasm. The partially double-stranded HBV genomic DNA (rcDNA) is converted to cccDNA, possibly by the host DNA repair mechanism, and then forms a mini-chromosome with the addition of nucleosomes, the HBV core protein and HBx. This mini-chromosome directs the synthesis of 3.5-kb pcRNA and pgRNA, 2.4-kb preS1 mRNA, 2.1-kb preS2/S mRNA, and 0.7-kb X mRNA, which serve as the templates for the synthesis of HBeAg, core proteins, polymerase, L, M, and S HBsAg proteins, and HBx, respectively. The pgRNA is packaged together with the DNA polymerase by the core protein to form the nucleocapsid. The reverse transcription ensues to convert the pgRNA into the rcDNA genome. The nucleocapsid may deliver the rcDNA back into the nucleus for the amplification of cccDNA. Alternatively, it may interact with HBsAg for envelopment and the formation of the mature HBV particle. Both multivesicular bodies (MVBs) and endosomal sorting complexes required for transport (ESCRT) are involved in the envelopment and the egress of HBV virions. In addition to complete virions, subviral particles (SVPs) consisting solely of HBsAg, empty virions, RNA-containing virions, and empty capsid particles are also released from HBV-infected cells.

Small Rab GTPases participate in the trafficking of late endosomes/MVBs and autophagosomes. One of the Rab proteins, Rab7, is activated by HBV and induces tubulation of MVBs and autophagosomes and their fusion with lysosomes, leading to the lysosomal degradation of HBV particles. The inhibition of Rab7 or lysosomal functions enhances HBV secretion[321]. Rab33B participates in the formation of autophagosomes via its interaction with the Atg5-Atg12-Atg16L1 complex and is required for the assembly or stability of the naked capsid particles and their egress[322]. Rab5B is required for the transport of the L surface protein from the ER to MVB, and its depletion results in the colocalization of the L protein with capsid particles in the ER, supporting the possible involvement of the ER and MVBs in the envelopment of HBV nucleocapsid particles and viral egress[323]. The lifecycle of HBV is summarized in Figure 4.

In contrast, HBV SVPs are formed on ER membranes and secreted from cells via the ER-Golgi secretory pathway [324,325], involving the COPII anterograde transport machinery[326]. The release of naked capsids depends on the Alix-assisted exocytosis but it does not require ESCRT, even though Alix is involved in the ESCRT-dependent exocytic pathway[327]. This release of naked capsid particles is also dependent on the Rab33B GTPase and its associated Atg5-Atg12-Atg16L1 complex, and does not appear to involve autophagosomes[328].

Finally, it had recently been shown that apolipoprotein E (ApoE) was associated with mature HBV virions but not naked capsid particles. This association is important for the production and the infectivity of HBV, as the silencing of ApoE in cells significantly suppressed the production of HBV and reduced its infectivity[329].

## HBV pathogenesis

### Risk factors for HBV-induced HCC

HBV is a major cause of HCC. In HBV endemic areas such as Asia-Pacific countries, approximately 80% of newly diagnosed HCC are related to chronic HBV infection[330]. Liver cirrhosis can significantly increase the risk for HCC in chronic HBV patients[331]. Other factors such as age, gender, the serum alanine aminotransferase (ALT) level, the serum HBV DNA level (i.e., viral load), and the HBeAg level can also affect the risk for HCC[332]. For example, regardless of the severity of liver cirrhosis, the combination of a high ALT level, HBeAg positivity, and a serum HBV DNA level of ≥10,000 copies/mL is considered as a strong predictor for the development of HCC [333–335]. High viral load by itself is also a predictor for the postoperative recurrence of HCC [336,337]. HCC also exhibits a gender disparity among HBV carriers with a male to female ratio of 5–7:1[338]. The same gender disparity of HCC incidence was also observed in HBV transgenic mice[339]. This gender disparity can be at least partially attributed to the positive feedback interaction between HBV and the androgen receptor (AR). HBx can bind to and enhance the activity of AR[249], or activate AR via the activation of Src and glycogen synthase kinase-β (GSK3β)[340], which can in turn activate HBV gene expression via its AREs located in the HBV genome to enhance HBV replication and hence its carcinogenesis [109,110]. The role of AR in HBV-induced hepatocarcinogenesis was confirmed in a study using HBV transgenic mice, in which it was shown that the liver-specific knockout of AR led to the reduction of HBV gene expression and HCC incidence[341]. There is also a gender disparity in the level of chronic liver inflammation, which also promotes HBV-induced hepatocarcinogenesis.

The integration of HBV DNA into the host chromosomes is found in more than 80% of HBV-associated HCC [342,343]. The most frequent integration sites in the HBV genome is in the HBx coding sequence, resulting in the generation of chimeric RNA transcripts with both host and HBV sequences and the expression of C-terminally truncated HBx [20,344,345]. The selection of the integration sites in the host chromosomes was initially thought to be random. However, more recent whole-genome sequencing studies led to the identification of recurrent integration hotspots[346], which include genes encoding the telomerase reverse transcriptase (TERT), the protein tyrosine phosphatase receptor type D (PTPRD), tumor protein 53 (TP53), retinoic acid receptor beta (RARB), catenin beta 1 (CTNNB1), etc[347]. The integration of HBV DNA may activate or disrupt the expression of these genes to cause host chromosome instability and promote cancer development, metastasis and angiogenesis. Readers are referred to our recent review for details on this topic[347].

### HBV genotypes and viral pathogenesis

Based on the genomic sequence, HBV has been grouped into ten genotypes named from A to J, and many more subtypes [348–350]. Different genotypes have distinct geographic distributions. For examples, genotype A is prevalent in western Africa, northern Europe, genotype D is widespread in Africa, Europe, India, and the Mediterranean region, and genotypes B and C are prevalent in Asia [351]. HBV genotypes can affect viral virulence, pathogenicity, clinical outcome, and response to type I interferon (IFN) therapies. Chronic HBV carriers infected by HBV genotypes C or D have lower rates of HBV DNA loss and HBeAg seroconversion in response to IFN treatment than patients infected by genotype A or B [352–354]. HBeAg seroconversion is the loss of HBeAg with the concomitant appearance of the anti-HBeAg antibody. It is often associated with the activation of the T cell response[355]. The infection by HBV subgenotype A2 is often associated with high viral load after the horizontal transmission of the virus[356].

Children chronically infected by HBV genotype A have lower viral load and less severe symptoms than children infected by genotype D [357,358]. Moreover, HBV genotypes A and D differ in their effects on liver pathogenesis [359,360] and resistance to the deoxycytidine analog lamivudine[361]. When genotype B and genotype C were compared, genotype C was found to have a higher frequency of HBeAg positivity and a higher serum level of HBV DNA than genotype B, and have a delayed HBeAg seroconversion in the immune clearance phase of chronic infection[362]. Genotype C is also associated with more severe liver diseases including cirrhosis and HCC, but genotype B is associated with the development of HCC in young patients with non-cirrhotic liver.

The infection by more than one HBV genotype can lead to genotypic recombination in patients. The mixed infection by genotype B and genotype C is correlated with a worse prognosis of the disease and higher viral load, comparing with the single infection by genotype C[363]. While there are differences in viral replication and pathogenesis among different HBV genotypes, the molecular basis underlying these differences remains unclear.

### HBV mutants and hepatocarcinogenesis

HBV DNA replication is error-prone because its polymerase lacks the proofreading activity with an error frequency of roughly one misincorporation for every 1600 nucleotides synthesized[364]. Naturally occurring nucleotide mutations have been found in all four HBV genes[365]. A double-nucleotide mutation of A to T at nt. 1762 (A1762T) and G to A at nt. 1764 (G1764T), which reside in the basal core promoter (BCP) and the X ORF, is frequently identified in patients with chronic hepatitis [366,367]. This double mutation converts a nuclear receptor binding site to the HNF1 binding site and reduced the pcRNA level without affecting the pgRNA level[368]. As such, it reduces specifically the expression of the precore protein and HBeAg without affecting the core protein level[366]. Notably, this BCP mutation is sometimes also associated with the G to A mutation at nt. 1896 (G1896A) in the precore sequence that abolishes the expression of HBeAg [369–371]. Both the BCP mutation and the G1896A mutation are associated with a high risk of HCC [372,373]. In one study, HBV patients with the T1762/A1764 mutation was found to develop HCC more frequently than those without with a odds ratio of 10.6 (*P* < 0.001)[374]. Other BCP mutations, such as T1753C and C1766T, either alone or in combination with other BCP mutations and/or the G1896A mutation, have also been found to be associated with a high incidence of HCC [360,375].

The A1762T and G1764A double mutation also changed amino acid 130 of the HBx sequence from lysine to methionine (K130M) and amino acid 131 from valine to isoleucine (V131I)[371]. This double amino acid substitution affects the biological activities of HBx and allows it to suppress the BCP activity[368]. Kwun and Jang also showed that HBx with the K130M mutation strongly inhibited the expression of cyclin-dependent kinase inhibitor p21 gene by suppressing the Sp1 TF activity[376]. In addition, HBV with the A1762T/G1764A/T1753A/T1768A quadruple mutation had also been shown to downregulate the expression of p53 and the S-phase kinase-associated protein 2 (Skp2) to promote the cell cycle[375]. HBx with the proline-38 to serine (P38S) mutation is also frequently identified in chronic HBV carriers with HCC and an independent risk factor for HCC[377] (*P* = 0.001, odds ratio: 4.89). These findings indicate that mutations in HBx can promote hepatocarcinogenesis.

Deletions and nucleotide substitutions are frequently detected in the S gene, causing mutations in HBsAg proteins [203,378]. This can lead to the development of ground-glass hepatocytes (GGHs), which is characterized by an abnormal formation of the ER and a liver pathology found in chronic hepatitis B patients [379,380]. The S gene mutants isolated from GGHs often have deletions in the S promoter region in the preS1 sequence and, as such, they express mostly the L HBsAg mutants with little M or S HBsAg, resulting in the retention of the L HBsAg mutants in the ER and the induction of the ER stress [381,382]. The ER stress can cause oxidative DNA damage and genome instability, leading to the development of HCC. In a clinical study, preS2-defective mutants were found to be more prevalent in chronic HBV carriers with HCC (84.2%) than in inactive HBV carriers without HCC (13.33%) or in carriers with cirrhosis (50%)[383]. Indeed, HBV with a mutation in the preS2 sequence had been shown to induce HCC in transgenic mice[339].

The study of the HBV polymerase also revealed that the mutations of A to G at nt. 799 (A779G), A to G at nt. 987 (A987G) and T to A at nt. 1055 (T1055A) in the coding sequence of the RT domain were independent risk factors for HCC, with adjusted odds ratios of 5.53, 4.20 and 3.78, respectively[384]. A longitudinal study indicated that these mutations could be detected 4–5 years before HCC diagnosis, supporting a causative role of these mutations in the development of HCC[384]. The A987G mutation does not alter the coding sequence of the RT domain. However, the A779G mutation converts isoleucine to valine and the T1055A mutation converts methionine to lysine. How these nucleotide changes increase the risk for HCC will require further research. In a separate study, three nucleotide mutations, T31C, G529A and T1078G in the polymerase gene, which all cause missense mutations, were identified as the predictor correlated with the postoperative survival of chronic HBV carriers with HCC[385]. The T31C mutation resides in the coding sequence of the spacer domain, and G529A and T1078G mutations reside in the coding sequence of the RT domain. How these mutations may affect the survival rate of HBV patients with HCC remains unclear.

### HBV spliced RNA variants and viral pathogenesis

In addition to HBV mRNAs, spliced RNA variants (spRNAs) derived from the pgRNA have also been found in hepatoma cell lines transfected with the HBV-expressing plasmids [386–388], HBV transgenic mice[389], and the liver tissues isolated from chronic hepatitis B (CHB) patients [390,391]. The spRNAs that may be generated differ between different HBV genotypes [392,393]. The study of a chronic HBV patient over a 15-year period following liver transplantation led to the identification of highly diverse and novel spRNA populations[394], indicating that the population of spRNAs can be highly dynamic during chronic HBV infection. The most abundant HBV spRNA variant, which may reach up to 30% of the pgRNA, is a 2.2-kb RNA molecule derived from the pgRNA by the removal of a 1.3-kb intron [390,395]. spRNAs can be packaged into capsid particles and reversed transcribed into spDNAs to generate defective HBV particles[396]. To date, the functions of spRNAs are still mysterious, as they are not essential for HBV replication in cultured cells and mice. HBV spRNAs can bind to TATA-box binding protein (TBP) and act as a repressor of HBV RNA transcription and may play a role in chronic HBV infection[397].

HBV spRNAs can also be translated to produce novel HBV proteins. HBV splice-generated protein (HBSP) is a major spRNA product[398]. It contains part of the polymerase sequence fused to a new ORF and can suppress the IFN-signaling pathway by inhibiting the phosphorylation of STAT1 and its subsequent nuclear translocation that is required for the activation of ISGs[395]. An N-terminally truncated polymerase, also a product of spRNA has a similar inhibitory effect on IFN signaling[395]. Indeed, the increase of a specific population of HBV spRNAs, including the spRNA encoding HBSP, in chronic hepatitis B patients had been shown to correlate with an impaired response to the IFN-α therapy[395]. HBSP can induce T-cell responses in a mouse model and in HBV-infected patients[399]. It can also suppress the NF-κB pathway activated by TNF-α and reduce the hepatic infiltration of immune cells during chronic liver inflammation in a mouse model[400], and suppress Fas-induced apoptosis via the activation of the PI3K-AKT pathway in hepatoma cells and primary human hepatocytes[401]. These findings together suggest an important role of HBSP in the survival and persistence of HBV-infected hepatocytes during chronic infection.

The 2.2-kb single-spliced RNA can also produce a p21.5-kDa protein that is one amino acid shorter than the core protein. p21.5 can form a homodimer that interacts with the core protein dimer and suppresses the formation of capsid particle [398,402]. spRNAs also regulate host immunity through their effect on the synthesis of chemokines in hepatocytes, which may promote liver immunopathogenesis, immune escape of HBV, and the progression of liver fibrosis during chronic HBV infection [403,404]. The 2.2-kb single-spliced RNA is present at a higher level in liver tumor tissues than in peri-tumor tissues, raising the possibility that this spliced RNA may be involved in the development of HCC[405]. Bayliss et al. also found that the serum spDNA level was higher in HCC patients than in control patients without HCC, and there was a strong correlation between serum spDNA levels and time to HCC diagnosis, again suggesting a role of spRNAs in hepatocarcinogenesis[406]. In conclusion, the studies on spRNAs and the proteins they encode indicate that they may regulate the crosstalk between HBV and hepatocytes to affect viral persistence and pathogenesis (for further details on the role of spRNA in HBV pathogenesis, please see the recent review[407]).

## HBV and liver immunopathogenesis

### HBV inoculum and type I IFNs

The ability of HBV to establish persistence is influenced by the size of the viral inoculum. By using chimpanzees as a model, Asabe et al. found that the infection of the animals with a low-dose HBV inoculum of 1 or 10 genome-equivalent (GE) would lead to the infection of 100% hepatocytes and viral persistence with severe immunopathology[408]. However, the infection of the animals with the inoculum of 10^4^ or 10^7^ GE led to the infection of fewer than 0.1% of hepatocytes and viral clearance with minimal immunopathology. Tian *et al*. also reported that a low-dose inoculation of HBV DNA into mice by hydrodynamic injection would prolong HBV persistence [409,410]. Their further studies indicated that this was due to the induction of the type I IFN response, which stimulated HBV gene expression and replication [409,410]. In contrast, they also found that type I IFNs suppressed HBV replication when viral load was high. IFN-α as well as IFN-γ can induce the expression of the human cytidine deaminase APOBEC3G [411,412], which binds to the viral polymerase and is thus incorporated into the nucleocapsid as well. The binding of APOBEC3G to the viral DNA polymerase interferes with the minus-stranded DNA synthesis [413,414]. The study of chronic HBV patients indicated that the induction of APOBEC3G might be mediated by the JAK-STAT signaling pathway[415]. These findings indicate that the size of the viral inoculum can have a profound impact on viral persistence or clearance and that type I IFNs can positively or negatively regulate HBV replication, depending on viral load.

### Cytotoxic T lymphocytes

HBV-specific CD8^+^ T cells recognize viral peptides presented by the major histocompatibility complex (MHC) class I molecules on the cell surface and play a crucial role in HBV clearance[416]. Indeed, an active HBV-specific CD8^+^ T cell response, which recognizes multiple HBV epitopes in polymerase, core and surface proteins[417], and can be detected in patients who recover from acute HBV infection[418], whereas hypo-responsiveness of HBV-specific CD8^+^ T cells, which have restricted epitope specificities, is a characteristic of chronic HBV infection [419,420]. The exhaustion of HBV-specific cytotoxic T lymphocyte (CTL) response is characterized by a low frequency of IFN-γ and granzyme production, impaired sensitivity to HBV-specific antigen stimulation, increased sensitivity to apoptosis induced by tumor necrosis factor-related apoptosis-inducing ligand (TRAIL), and upregulation of the Inhibitory checkpoint receptors, etc [421,422]. Multiple coinhibitory checkpoint receptors, such as cluster of differentiation 244 (CD244/2B4), T cell immunoglobulin and mucin-domain containing-3 (Tim-3), cytotoxic T lymphocyte-associated antigen-4 (CTLA-4), and Program death-1 (PD-1) are overexpressed on the surface of exhausted T cells during chronic viral infection[422]. The enhanced expression of these immune checkpoint inhibitory receptors of T cells impairs the HBV-specific CTL response and plays an important role in T cell exhaustion in chronic HBV patients [423,424] and mouse models[423]. Maier *et al*. found that HBV-specific CTLs adoptively transferred into the liver of HBV transgenic mice failed to produce IFN-γ[425]. Notably, the blockade of the interaction between PD-1 and its ligand, Programmed death-ligand 1 (PD-L1), promoted the production of IFN-γ in the liver by these CTLs.

By studying mice with persistent HBV replication, Tian *et al*. found an increased expression of PD-1 in HBV-specific CD8^+^ T cells, which had impaired response to HBV[159]. Their further analysis also revealed an increased expression of PD-L1 in Kupffer cells, the resident macrophages of the liver. The CD8^+^ T cell activity could be restored by treating the mice with a specific antibody directed against PD-L1 or by the depletion of Kupffer cells, leading to HBV clearance. Their studies demonstrated an important role of the interaction between PD-1 and PD-L1 in the suppression of HBV-specific CTL response and HBV persistence. The B7 homolog 3 (B7-H3; a.k.a. CD276) is another member of the B7 family that was originally identified as a costimulatory molecule that induces T-cell proliferation and IFN-γ production *in vitro*[426]. Luan *et al*. found that the level of soluble B7-H3 increased in the plasma of patients with chronic HBV infection, and this increase was accompanied by the reduced level of membrane-associated B7-H3 on hepatocytes[427]. Their further studies indicated that soluble B7-H3 could partially block membrane-associated B7-H3 from inhibiting the T cell receptor-induced proliferation of T cells and the secretion of IFN-γ. This effect of soluble B7-H3 likely contributes to the activation of the CTL response and the induction of hepatic inflammation in chronic HBV patients.

CD8^+^ T cell-derived IFN-γ plays a key role in the progression of chronic liver diseases by recruiting and activating macrophages to produce fibrosis-promoting cytokines and chemokines such as TNF-α, IL-6, and MCP-1^431^. Note that, in addition to HBV-specific T cells, it had also been shown that many virus non-specific T cells frequently infiltrated the liver of CHB patients[428] and HBV transgenic mice [429], and contribute to the progression of chronic liver diseases.

### Antibody-dependent cell-mediated cytotoxicity

In addition to CTL-mediated immune responses, an antibody-dependent cell-mediated cytotoxicity (ADCC) is also involved in liver pathogenesis during acute and chronic active hepatitis B[430]. Peripheral blood B lymphocytes isolated from chronic HBV patients with active hepatitis can induce significant cytotoxicity on hepatocytes [431–433]. The recognition of viral antigens present on the target cell surface by the viral antigen-specific antibodies is a crucial step for the initiation of ADCC. Fc receptors expressed on immune effector cells will then recognize the Fc domain of the antibodies and stimulate the release of cytotoxic granules to eliminate the target cells [434]. HBsAg, HBeAg and HBcAg can be detected on the hepatocellular membrane of chronic HBV patients [435,436], and the anti-HBcAg have also been detected on the hepatocellular membrane[437]. Michalak et al. found that the anti-HBcAg-induced cytotoxicity was significantly enhanced in chronic HBV patients with active hepatitis than those without, and anti-HBsAg could also induce hepatocytotoxicity in the presence of complement[438]. Their results indicated that HBcAg and HBsAg on the hepatocellular surface could be recognized by their respective antibodies, and that the complement-dependent cytolysis could contribute to liver injury caused by HBV infection.

### Natural killer cells and regulatory T cells

Natural killer (NK) cells are key mediators of the ADCC response and play an important role in the early control of HBV infection [439,440]. They can also directly respond to viral infections by secreting cytokines including TNF-α, IL-10, and IFN-γ and by releasing cytotoxic mediators such as granzyme and perforin [441,442]. Recent studies using a mouse model mimicking acute HBV infection indicated that NK cells could promote the CTL response against HBV via the producing IFN-γ[443]. However, in chronic HBV patients, peripheral NK cells are functionally impaired, have reduced ability to produce IFN-γ and TNF-α, and display molecular features, such as the upregulation of immune checkpoints receptors and their ligands, typical of those observed in exhausted T cells, without the reduction of their cytolytic activity [444–448]. The activity of NK cells can be modulated by the expression of activating or inhibitory receptors on the cell surface[449], and a decreased expression of the activating receptors CD16, NKp30, NKG2D and 2B4, and the increased expression of the inhibitory receptor NKG2A are observed in chronic HBV patients [444,446]. A recent study revealed that dysfunctional NK cells in chronic HBV patients had enriched RNA transcripts that were also expressed in exhausted T cells, suggesting a common mechanism for NK cell dysfunction and T cell exhaustion[444]. Further studies indicated that this might be due to the dysregulation of calcium signaling in both dysfunctional NK cells and exhausted T cells, which led to the activation of the transcription factor TOX and several of its target genes in a pathway dependent on the calcium-associated transcription factor NFAT[444]. In addition, high levels of transforming growth factor-β1 (TGF-β1) had also been shown to suppress the proliferation of NK cells *ex vivo* and were detected in the sera of chronic HBV patients[446]. TGF-β1 downregulated the expression of NKG2D and 2B4 in NK cells and induced the cell cycle arrest. It likely also plays an important role in the suppression of NK cell activity. Interestingly, NK cells were found to eliminate HBV-specific CD8 + T cells in a contact-dependent manner and their depletion from the peripheral blood mononuclear cells (PBMCs) of chronic HBV patients augmented HBV-specific CD8 + T cell responses *ex vivo*[450]. NK cells are the main intrahepatic lymphocytes that express TRAIL, allowing them to kill hepatocytes bearing TRAIL receptors, leading to chronic liver injury[448]. Intrahepatic CD8 + T cells in chronic HBV patients also express a high level of TRAIL death receptor R2 (TRAIL-R2), which sensitizes them to NK cell-induced apoptosis[450]. These studies underscore the importance of NK cells in HBV persistence.

Regulatory T cells (Tregs) are a specialized subpopulation of T cells that can suppress effector T cells via the secretion of inhibitory cytokines[451]. In chronic HBV patients, there is an increased frequency of circulating Tregs (CD4^+^CD25^+^FoxP3^+^) [452–454]. In a recent study, it was also found that IL-10 secreted by the circulating Tregs could also contribute to the dysfunction of NK cells in chronic HBV patients[455]. It was found that HBeAg could stimulate Tregs to produce IL-10, thereby increasing the expression of NKG2A in NK cells and contributing to the dysfunction of NK cells in chronic HBV patients. Notably, the HBV-specific T cell proliferation in response to PD-1 blockade was weaker in HBeAg-positive patients than in HBeAg-negative patients, in support of a role of HBeAg in T cell exhaustion and immune tolerance[454].

### Macrophages

Kupffer cells comprise approximately 15% of the total liver cell population[456]. They are derived from the yolk sac, mature in the fetal liver and retain their hepatic residence after birth and during adulthood[457]. In a study using transgenic mice carrying the replication-competent HBV DNA genome as a model, Sitia et al. found that the depletion of Kupffer cells before the adoptive transfer of HBV-specific CD8^+^ T cells exacerbated liver injury caused by these transferred CD8^+^ T cells[458]. Their further studies indicated that Kupffer cells did not directly affect the function and the pathogenic potential of HBV-specific CD8^+^ CTLs, but rather, their depletion led to impaired removal of apoptotic hepatocytes and the development of focal hepatocellular necrosis, which exacerbated the liver injury due to the release of damage-associated molecular patterns (DAMPs) and the infiltration of inflammatory cells[458]. Their results indicated that Kupffer cells could limit immunopathology of the liver by removing apoptotic hepatocytes. Kupffer cells, like other macrophages, can undergo the proinflammatory M1 polarization characterized by the expression of proinflammatory cytokines such as TNF-α and IL-1β, or the M2 anti-inflammatory polarization characterized by the expression of IL-10 and arginase-1.

The C3H/HeN mouse strain is tolerant to HBV at 6 weeks of age but not at 12 weeks of age, when the HBV genomic DNA cloned in an adenovirus-associated virus (AAV) vector was introduced into mouse hepatocytes using hydrodynamic injection. By using this approach, Wu et al. found that 12-week-old mice injected with the HBV genomic DNA had a higher level of TNF-α secreting Ly6C^+^ monocytes and a lower level of IL-10-secreting Kupffer cells in the liver at 3 days after DNA injection than 6-week-old mice[459]. Their further analysis indicated that the enhanced recruitment of Ly6C^+^ monocytes to the liver was due to the increased secretion of the chemokine CCL2 by hepatocytes. They further showed that the treatment of mice with an antagonist to the C-C chemokine receptor 2 (CCR2) would hamper the recruitment of Ly6C^+^ monocyte to the liver and delay HBV clearance in 12-week-old mice, and the depletion of Kupffer cells would enhance the recruitment of Ly6C+ monocytes and accelerate HBV clearance. Their results revealed opposite roles of Ly6C+ monocytes and Kupffer cells in the control of HBV clearance or persistence, and the relative populations of these two cell types in the liver were affected by the mouse age[459]. By using a similar approach, Li et al. found that the expression of TLR2 was upregulated in Kupffer cells of mice with persistent HBV replication, and the activation of TLR2 with HBcAg enhanced the expression of IL-10 in these Kupffer cells[460,461]. They also found that the knockout of TLR2 or the depletion of Kupffer cells led to the activation of CD8^+^ T cells and the elimination of HBV from mice. Their results were consistent with the finding of Wu et al. and supported a role of Kupffer cells in HBV persistence.

Tian et al. also studied Kupffer cells using adult C57BL/6 mice as a model. In contrast, they found that Kupffer cells isolated from naïve mice would undergo the M1 polarization when stimulated with HBV in the presence of HBeAg, whereas Kupffer cells isolated from HBV-negative mice born to replication-competent, HBeAg^+^, hemizygous HBV transgenic dams would undergo the M2 polarization under the same treatment. They further found that M2 Kupffer cells expressed a high level of PD-L1 and could suppress HBV-specific CD8 + T cells to promote HBV persistence, and their depletion could lead to HBV clearance from mice. Their results indicated that Kupffer cells can have both anti- and pro-HBV effects, depending on whether they had been conditioned by maternal HBeAg[159].

In yet another study using immunodeficient mice grafted with human hematopoietic stem cells and liver progenitor cells for the reconstitution of human immune system and liver cells, it was found that 75% of mice infected by HBV led to HBV persistence and had impaired human immune responses in the liver and developed liver fibrosis. Importantly, these mice also had a high level of infiltrated macrophages with M2-like phenotype[461]. These results together indicate that Kupffer cells can limit liver injury induced by HBV and play a positive or negative role in HBV clearance and HBV-induced liver pathogenesis, depending on microenvironmental cues (e.g., HBeAg).

**Figure 5.**
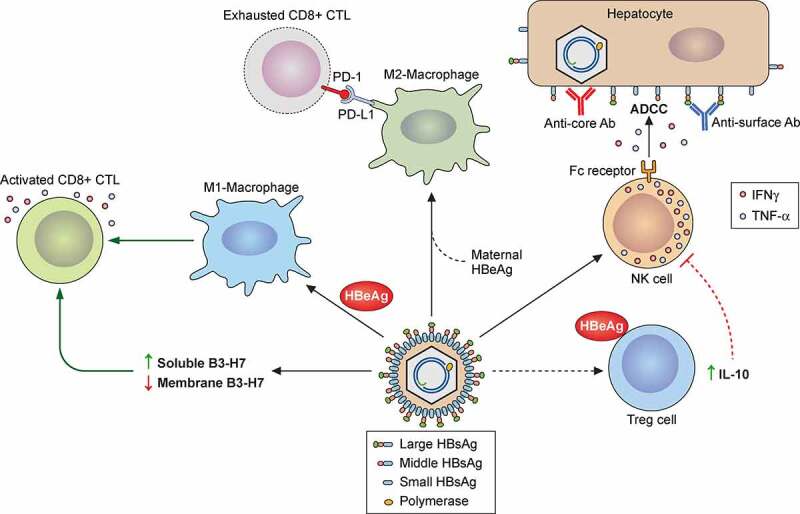
**Roles of immune cells in HBV-induced liver pathogenesis**. HBV particles and HBeAg can activate hepatic macrophages (Kupffer cells) to undergo the pro-inflammatory M1 polarization, which subsequently express TNF-α and IL-1β and stimulate HBV-specific CTLs for HBV clearance. Alternatively, HBV in the presence of HBeAg can also stimulate hepatic macrophages that have been conditioned by maternal HBeAg to undergo the anti-inflammatory M2 polarization with an increased expression of PD-L1. The binding of PD-L1 to PD-1 on HBV-specific CTLs can lead to T cell exhaustion and HBV persistence. In chronic HBV patients, an elevated level of B7-H3 in the plasma can impair the activity of HBV-specific CTLs. HBV replication can activate NKs to produce IFN-γ and TNF-α to suppress HBV replication. Moreover, the Fc receptor of NKs can recognize anti-surface or anti-core antibodies bound to the surface of HBV-infected hepatocytes to trigger the ADCC during acute and chronic HBV infection. HBeAg can also stimulate Tregs to express IL-10 to cause the dysfunction of NK cells and the establishment of immune tolerance during chronic HBV infection.

A summary of immune cells that play a role in HBV persistence and clearance is illustrated in Figure 5.

## Data Availability

Data sharing is not applicable to this article as no new data were created or analyzed in this review.
